# Applying Andersen's healthcare utilization model to assess factors influencing patients' expectations for diagnostic tests at emergency department visits during the COVID-19 pandemic

**DOI:** 10.3389/fpubh.2023.1250658

**Published:** 2023-11-23

**Authors:** Zhilian Huang, Karthiga Natarajan, Hoon Chin Lim, Yanyi Weng, Hann Yee Tan, Eillyne Seow, Li Lee Peng, Jing Teng Ow, Win Sen Kuan, Angela Chow

**Affiliations:** ^1^Infectious Diseases Research and Training Office, National Centre for Infectious Diseases, Singapore, Singapore; ^2^Department of Preventive and Population Medicine, Office of Clinical Epidemiology, Analytics, and Knowledge [OCEAN], Tan Tock Seng Hospital, Singapore, Singapore; ^3^Department of Accident and Emergency, Changi General Hospital, Singapore, Singapore; ^4^Department of Emergency Medicine, Tan Tock Seng Hospital, Singapore, Singapore; ^5^Department of Acute and Emergency Care, Khoo Teck Puat Hospital, Singapore, Singapore; ^6^Department of Emergency Medicine, National University Hospital, Singapore, Singapore; ^7^Department of Surgery, Yong Loo Lin School of Medicine, National University of Singapore, Singapore, Singapore; ^8^Lee Kong Chian School of Medicine, Nanyang Technological University, Singapore, Singapore; ^9^Saw Swee Hock School of Public Health, National University of Singapore, Singapore, Singapore

**Keywords:** emergency medicine, upper respiratory tract infection (URTI), COVID-19, diagnostic services, pandemic (COVID-19), emergency department (ED) utilization

## Abstract

**Background:**

The uncertainties surrounding the COVID-19 pandemic led to a surge in non-urgent emergency department (ED) attendance among people presenting with upper respiratory tract infection (URTI) symptoms. These non-urgent visits, often manageable in primary care, exacerbated ED overcrowding, which could compromise the quality of ED services. Understanding patients' expectations and the reasons for these ED visits is imperative to mitigate the problem of ED overcrowding. Hence, we assessed the factors influencing patients' expectations for diagnostic tests during their ED visits for uncomplicated URTI during different phases of the pandemic.

**Methods:**

We conducted a cross-sectional study on adults with URTI symptoms seeking care at four public EDs in Singapore between March 2021 and March 2022. We segmented the study period into three COVID-19 pandemic phases—containment, transition, and mitigation. The outcome variables are whether patients expected (1) a COVID-19-specific diagnostic test, (2) a non-COVID-19-specific diagnostic test, (3) both COVID-19-specific and non-COVID-19-specific diagnostic tests, or (4) no diagnostic test. We built a multinomial regression model with backward stepwise selection and classified the findings according to Andersen's healthcare utilization model.

**Results:**

The mean age of participants was 34.5 (12.7) years. Factors (adjusted odds ratio [95% confidence interval]) influencing expectations for a COVID-19-specific diagnostic test in the ED include younger age {21–40 years: (2.98 [1.04–8.55])}, no prior clinical consultation (2.10 [1.13–3.89]), adherence to employer's health policy (3.70 [1.79–7.67]), perceived non-severity of illness (2.50 [1.39–4.55]), being worried about contracting COVID-19 (2.29 [1.11–4.69]), and during the transition phase of the pandemic (2.29 [1.15–4.56]). Being non-employed influenced the expectation for non-COVID-19-specific diagnostic tests (3.83 [1.26–11.66]). Factors influencing expectations for both COVID-19-specific and non-COVID-19-specific tests include younger age {21–40 years: (3.61 [1.26–10.38]); 41–60 years: (4.49 [1.43–14.13])}, adherence to employer's health policy (2.94 [1.41–6.14]), being worried about contracting COVID-19 (2.95 [1.45– 5.99]), and during the transition (2.03 [1.02–4.06]) and mitigation (2.02 [1.03–3.97]) phases of the pandemic.

**Conclusion:**

Patients' expectations for diagnostic tests during ED visits for uncomplicated URTI were dynamic across the COVID-19 pandemic phases. Expectations for COVID-19-specific diagnostic tests for ED visits for uncomplicated URTI were higher among younger individuals and those worried about contracting COVID-19 during the COVID-19 pandemic. Future studies are required to enhance public communications on the availability of diagnostic services in primary care and public education on self-management of emerging infectious diseases such as COVID-19.

## Introduction

Hospital emergency departments (EDs) are vital components of the healthcare system as they provide immediate acute care for patients presenting with urgent medical conditions that may be life-threatening (1). However, growing reliance on emergency services has resulted in overcrowding in EDs globally (2, 3). Non-urgent ED attendances, which account for 9%−60% of ED visits, are among the main contributors to the problem of overcrowding in EDs (4, 5). Medical conditions that are manageable in the primary care setting account for a substantial proportion of non-urgent visits to the ED, resulting in overcrowding, long waiting times, increased healthcare-associated costs, high staff burden, and suboptimal use of hospital resources (2, 6). The quality of emergency services will be compromised if the rising numbers of non-urgent visits further strain the already overworked ED staff (2, 7, 8).

Patients seek non-urgent care in the ED for a myriad of reasons. These reasons include lack of access to primary healthcare facilities, lack of diagnostic services in primary care, referrals from primary care facilities, easy access to EDs, patient perceptions regarding the severity of their condition, and perceptions of better care in EDs (2, 3, 5, 9, 10). Efforts to mitigate ED overcrowding, such as teleconsultation, education on appropriate usage of ED services, and improving primary care access, have shown mixed results (11–13).

Despite modest success in efforts to alleviate the patient load pressure in the ED, the unprecedented coronavirus disease 2019 (COVID-19) pandemic has changed the health-seeking behavior of the public (14). The uncertainties surrounding the pandemic led to a surge in the number of patients presenting with URTI symptoms seeking emergency care worldwide, including Singapore (15–17). The emergency departments in public hospitals experienced a surge in demand regarding COVID-19-related issues, leading to healthcare worker fatigue and longer wait times (18, 19). Though these visits were acceptable during the initial stages of the pandemic, their urgency and relevance were reduced as the majority of the population was vaccinated through the National Vaccination Programme in Singapore (20). From 15 September 2021, the Ministry of Health advised low-risk vaccinated individuals diagnosed with COVID-19 to recover from home through the Home Recovery Programme or seek care from primary care facilities. Pre-pandemic, patients with uncomplicated URTI usually seek care in government-funded or private primary care clinics in their neighborhood to obtain a diagnosis. ED services are reserved for intermediate- to high-risk patients requiring urgent acute care (21).

Although the non-severity of COVID-19 among the highly vaccinated population rendered ED visits for uncomplicated URTI symptoms unnecessary, patients presenting with suspected COVID-19 and uncomplicated URTI symptoms continued to seek medical care at EDs in Singapore. The lack of awareness of national protocols devised for the public on self-management and the appropriate channels for seeking care, coupled with anxiety associated with contracting COVID-19, could have resulted in such ED visits (15).

With the evolving COVID-19 pandemic affecting the heath-seeking behavior of the public, it is imperative to understand patients' expectations and the reasons for these ED visits to optimize ED resources and design interventions that can reduce inappropriate ED visits. Anchoring on Andersen's healthcare utilization model, we assessed the factors influencing patients' expectations for diagnostic tests during their ED visits for uncomplicated URTI during the different phases of the pandemic.

## Methods

### Study design and setting

We conducted a cross-sectional survey on adults seeking medical care in the ED for uncomplicated URTI during the COVID-19 pandemic. Our study covered the EDs of four acute public hospitals, constituting 40% of the public hospitals in Singapore. Given the dynamic situation of the pandemic, we segmented the study period into three phases.

#### Phase 1: containment (15 March 2021–7 May 2021)

The containment phase includes the period when Singapore adopted a zero COVID-19 policy. All known COVID-19-positive cases were isolated, and the authorities conducted extensive contact tracing to break possible chains of transmissions.

#### Phase 2: transition (8 May 2021–31 August 2021)

The transition phase was when the Delta wave occurred in the community. The Delta strain's higher transmissibility rendered contact tracing and containment impractical. On 8 May 2021, the Multi-Ministry Taskforce announced new measures to bring down the rates of COVID-19 community transmission.

#### Phase 3: mitigation (1 September 2021–2 March 2022)

As vaccination coverage in the population increased to >70%, safe COVID-19 management measures were relaxed, and the COVID-19 home recovery program was introduced to ease the demand for healthcare services. We termed this phase “mitigation” as the government announced in September 2021 that each household would receive free antigen rapid test (ART) kits to self-manage COVID-19 at home.

### Participants

We recruited adults who attended the EDs between March 2021 and March 2022. Patients must be diagnosed with an uncomplicated URTI (ICD-10 J00-J06) to meet the study inclusion criteria. We excluded patients admitted to the hospital and those with prior attendances to the ED within 30 days to avoid recruiting patients with complicated URTIs. COVID-19 suspects, identified through triage, were initially excluded from the study due to a default hospitalization policy. We included these patients from July 2021 onward following the revision of the national policy which encouraged home recovery for COVID-19 patients when more than 70% of the population had received at least one dose of the COVID-19 vaccination. A hospital admission was unnecessary when the presentation of COVID-19 symptoms became milder in the patient population. Consecutive patients were screened and monitored at triage; eligible patients were approached and recruited only after their medical consultation at the point of discharge from the emergency department.

### Questionnaire

The questionnaire was interviewer-administered by trained data collectors to enable consistency across data collection. We validated the questionnaire with emergency physicians and other healthcare professionals and piloted the questionnaire in the ED to ensure its viability. In addition to collecting the patient's demographics (age, gender, race, nationality, and education level), we collected information on the health status of patients (smoking status and Charlson's co-morbidity index) and factors that could be associated with their health-seeking behavior (reasons for the ED visit, any prior healthcare consultation for URTI, employment status, and payment method). Participants can select the reasons for their ED visit from a list or indicate additional reasons as free text. The list of reasons for ED visits is shown in Table 1.

**Table 1:** Classification of data variables and reasons for visiting the ED according to Andersen's healthcare utilization model.

**Andersen's category**	**Model variables**	
Predisposing factors	Age	
	Gender	
	Race	
	Nationality	
	Education	
	Charlson's comorbidity severity	
	Smoking status	
	**Reasons for visiting the ED**	
	Advised by family/friends/colleagues to visit the ED	• My family member/friend/colleague/other people (not doctor) advised me to seek care at the emergency department.
	Trust that ED is high quality and thinks it is better than primary care clinics	• I think that the care that I will receive from the emergency department for my illness is better than that from GP clinics/polyclinics. • I trust the quality of care that this emergency department provides. • I want a more thorough checkup for my current illness. • Faster treatment
Enabling factors	Employment status	
	Payment method	
	**Reasons for visiting the ED**	
	Bill covered by an employer	• The medical bill for the emergency department visit is covered by my employer/insurance.
	Adhering to the employer's policy	• My company requires me to obtain a medical certificate from a public healthcare institution. • URTI protocol for healthcare workers • Full-time national servicemen URTI protocol.
	Referred by healthcare provider	• Singapore Civil Defense Force ambulance • Referred by specialists/other clinics. • Ministry of Health's advice on COVID
	Convenient	• I live/work close (within 3 km) to this emergency department. • This emergency department is open 24 h, and I can attend at my convenience/clinic closed. • I have previously attended/been admitted to this hospital and have medical records here.
Individual needs	**Reason for visiting the ED**	
	Having persistent symptoms/conditions for their illness	• I have a persistent cough/runny nose/sore throat/other respiratory symptoms. • I have a persistent fever. • My condition has not improved although I have consulted a GP/polyclinic doctor.
	Thinks that illness is severe	• My cough/runny nose/sore throat/other respiratory symptoms are very severe. • I am worried that I may have dengue. • I am worried I may have a serious infection. • I am worried I may have a serious disease. • I have a very high fever.
	Worried about contracting COVID-19	• I am worried that I may have COVID-19.

### Outcomes

There were four outcome variables for this study: whether patients expected (1) a COVID-19-specific diagnostic test, (2) a non-COVID-19-specific diagnostic test (i.e., blood test, flu virus test, or chest X-ray), (3) both COVID-19-specific and non-COVID-specific diagnostic tests, or (4) no diagnostic test. COVID-19-specific diagnostic tests included SARS-CoV-2 antigen rapid test or polymerase chain reaction test.

### Classification with Andersen's healthcare utilization model

We employed Andersen's healthcare utilization model to analyze the drivers of ED visits for uncomplicated URTI (22). This model describes the utilization of healthcare services as a function of three core factors:

(1) Predisposing factors—demographic and psychosocial features influencing health-seeking behavior,
(2) Enabling factors—factors facilitating access to healthcare services, and
(3) Individual needs—an individual's perception regarding his/her health condition and need for health services.

We classified demographic characteristics (age, gender, race, and nationality), health status of individuals, and psychosocial factors (advised by loved ones to visit ED, trust in ED's quality of care) under predisposing factors as these factors have an impact on an individual's health-seeking behavior. Factors that facilitated the utilization of ED services by patients were classified under enabling factors, and patients' concerns about their health condition were classified under individual needs. The reasons for visiting the ED were grouped to reduce the number of variables.

### Analysis

We first performed univariate analyses on each independent variable to assess the differences between the outcome categories. Categorical variables were assessed using Pearson's chi-square test, while categories with a small number of variables were assessed using the Fisher–Freeman–Halton exact test. Continuous variables were assessed using non-parametric tests.

Next, we used the backward stepwise selection method to build a multinomial regression model. The initial model included variables with p < 0.25 from the univariate analyses. Variables were individually dropped from the model if it resulted in a lower Akaike's information criteria and/or Bayesian information criteria value. The Charlson's co-morbidity index (CCI) was computed and classified into three categories (no co-morbidity—CCI 0, mild—CCI 1-2, moderate/severe—score CCI > 2) (23).

In addition, we present a bar chart to list the reasons our participants attend the ED. All analyses were performed with Stata version 15.0 (StataCorp LP, College Station, TX) and RStudio version 2022.02.3 (RStudio, PBC, Boston, MA).

### Ethical approval

This study was approved by the National Healthcare Group Domain Specific Review Board in Singapore. NHG DSRB Ref: 2019/00174. Written consent was sought from participants for participating in this study.

## Results

We screened 5,319 patients who visited the ED, of whom 1,234 were eligible. Of those eligible for the study, 683 (56%) consented to our study. Two participants were later excluded from the study as they did not meet the inclusion criteria after subsequent changes to their clinical statuses. We eventually analyzed the data of 681 participants.

### Baseline characteristics and univariate analyses of respondents

The baseline characteristics and univariate analyses of participants' expectations for diagnostic tests are shown in Table 2.

**Table 2:** Baseline characteristics and univariate analysis of patients by expectation for diagnostic services in the ED.

**Baseline characteristics of respondents, n (%)**	**All patients**	**Not expecting a diagnostic test**	**Expects a COVID-19-specific test**	**Expects a non-COVID-19-specific test**	**Expects COVID-19-specific + non-COVID-19-specific tests**	***P*-value**
	**(*****N*** = **681)**	**(*****N*** = **73)**	**(*****N*** = **296)**	**(*****N*** = **74)**	**(*****N*** = **238)**	
**Predisposing factors**	**n (%)**	**n (%)**	**n (%)**	**n (%)**	**n (%)**	
Age, mean (Min, Max)	34.5 (21, 88)	38.2 (21, 77)	31.1 (21, 75)	40.4 (21, 73)	35.1 (21, 88)	< 0.001^∧^
Aged 21–40	517 (75.9%)	48 (65.8%)	252 (85.1%)	45 (60.8%)	172 (72.3%)	
Aged 41–60	123 (18.1%)	16 (21.9%)	34 (11.5%)	16 (21.6%)	57 (23.9%)	
Aged above 60	41 (6.0%)	9 (12.3%)	10 (3.4%)	13 (17.6%)	9 (3.8%)	
Male	339 (49.8%)	38 (52.1%)	154 (52.0%)	34 (45.9%)	113 (47.5%)	0.640
**Race**						
Chinese	314 (46.1%)	35 (47.9%)	146 (49.3%)	29 (39.1%)	104 (43.7%)	0.745
Malay	174 (25.6%)	18 (24.7%)	65 (22.0%)	21 (28.4%)	70 (29.4%)	
Indian	114 (16.7%)	12 (16.4%)	51 (17.2%)	15 (20.3%)	36 (15.1%)	
Other races	79 (11.6%)	8 (11.0%)	34 (11.5%)	9 (12.2%)	28 (11.7%)	
**Nationality**						
Singaporean	498 (73.1%)	49 (67.1%)	225 (76.0%)	52 (70.3%)	172 (72.3%)	0.303
Permanent resident	68 (10.0%)	8 (11.0%)	32 (10.8%)	5 (6.8%)	23 (9.7%)	
Others	115 (16.9%)	16 (21.9%)	39 (13.2%)	17 (23.0%)	43 (18.1%)	
**Tertiary education**	224 (32.9%)	20 (27.4%)	91 (30.7%)	28 (37.8%)	85 (35.7%)	0.348
**Charlson's comorbidity severity**						
No comorbidity	621 (91.2%)	64 (87.7%)	282 (95.3%)	61 (82.4%)	214 (90.3%)	0.021^#^
Mild	52 (7.6%)	8 (11.0%)	13 (4.4%)	11 (14.9%)	20 (8.4%)	
Moderate/Severe	8 (1.2%)	1 (1.4%)	1 (0.3%)	2 (2.7%)	4 (1.7%)	
**Smoker**	149 (21.9%)	23 (31.5%)	65 (22.0%)	10 (13.5%)	51 (21.4%)	0.071
**Prior (Non-ED) consult for URTI**	207 (30.4%)	34 (46.6%)	51 (17.2%)	37 (50.0%)	85 (35.7%)	< 0.001
**Reason**: Advised by family/friends/colleagues to visit the ED	192 (28.2%)	15 (20.5%)	96 (32.4%)	17 (23.0%)	64 (26.9%)	0.115
**Reason**: Trust that ED is high quality and thinks it is better than primary care clinics	358 (52.6%)	38 (52.1%)	144 (48.6%)	41 (55.4%)	135 (56.7%)	0.294
**Enabling factors**						
**Payment method**	(***N*** = 676)	(***N*** = 72)	(***N*** = 295)	(***N*** = 73)	(***N*** = 236)	
Employee benefits	404 (59.8%)	28 (38.9%)	218 (73.9%)	27 (37.0%)	131 (55.5%)	< 0.001^#^
Government/private insurance	54 (8.0%)	9 (12.5%)	13 (4.4%)	10 (13.7%)	22 (9.3%)	
Out-of-pocket	209 (30.9%)	33 (45.8%)	62 (21.0%)	35 (47.9%)	79 (33.5%)	
Social subsidies	9 (1.3%)	2 (2.8%)	2 (0.7%)	1 (1.4%)	4 (1.7%)	
**Employment**						
Employed	511(75.0%)	60 (82.2%)	201 (67.9%)	55 (74.3%)	195 (81.9%)	< 0.001
Not employed	60 (8.8%)	7 (9.6%)	16 (5.4%)	17 (23.0%)	20 (8.4%)	
NSF	110 (16.2%)	6 (8.2%)	79 (26.7%)	2 (2.7%)	23 (9.7%)	
**Reason** Bill covered by an employer	195 (28.6%)	12 (16.4%)	111 (37.5%)	16 (21.6%)	56 (23.5%)	< 0.001
**Reason**: Adhering to the employer's health policy	300 (44.1%)	12 (16.4%)	170 (57.4%)	18 (24.3%)	100 (42.0%)	< 0.001
**Reason**: Referred by healthcare provider	158 (23.2%)	23 (31.5%)	42 (21.4%)	29 (39.2%)	64 (26.9%)	< 0.001
**Reason**: Convenient	466 (68.4%)	46 (63.0%)	220 (74.3%)	51 (68.9%)	149 (62.6%)	0.023
**Individual needs**						
**Reason:** Having persistent symptoms/conditions for their illness	266 (39.1%)	31 (42.4%)	99 (33.4%)	29 (39.2%)	107 (45.0%)	0.051
**Reason:** Thinks that illness is severe	289 (42.4%)	34 (46.6%)	96 (32.4%)	40 (54.1%)	119 (50.0%)	< 0.001
**Reason:** Worried about contracting COVID-19	210 (30.8%)	13 (17.8%)	94 (31.8%)	9 (12.2%)	94 (39.5%)	< 0.001
**COVID-19 phases**						
Containment	255 (37.4%)	37 (50.7%)	105 (35.5%)	34 (46.0%)	79 (33.2%)	< 0.01
Transition	275 (40.4%)	16 (27.4%)	145 (49.0%)	21 (28.4%)	93 (39.1%)	
Mitigation	151 (22.2%)	20 (27.4%)	46 (15.5%)	19 (25.7%)	66 (27.7%)	

^#^Fisher–Freeman–Halton test.

^∧^Kruskal–Wallis test.

NSF, Full-time national servicemen.

#### Predisposing factors

The mean age of participants was 34.5 (Min: 21, Max: 88) years. Participants expecting only a COVID-19 diagnostic test had the lowest mean age (31.1 years), while those expecting non-COVID-specific diagnostic tests had the highest mean age (40.4 years). Half of the participants were men (49.8%), 46.1% were of the Chinese race, 73.1% were Singaporeans, and 32.9% had tertiary education (bachelor's degree and above). Most participants (91.2%) had no pre-existing co-morbidities, 78.1% were non-smokers, and a third (30.4%) had a prior (non-ED) consult for URTI. A significantly lower proportion of participants (17.2%) who expected only the COVID-19 test had a prior healthcare consult for the same episode of illness compared with other groups (*p* < 0.001). Half of the participants (52.6%) visited the ED because they trusted the ED to provide high-quality care and felt that ED care was better than primary care, and 28.2% reported that their close contacts (i.e., family, friends, and colleagues) advised them to seek care in the ED.

#### Enabling factors

Three-quarters (75.0%) of the participants were employed, and 16.2% were full-time national servicemen. A significantly higher proportion (82.2%) of employed participants were expecting only a COVID-19 diagnostic test (*p* < 0.001). Of the types of healthcare financing, 59.8% had employee benefits, 30.9% had to pay out-of-pocket, and the rest had some form of insurance or subsidy. A significantly higher proportion (73.9%) of participants with employee healthcare benefits were expecting only a COVID-19 diagnostic test (*p* < 0.001).

We grouped participants into three COVID-19 phases based on the date they visited the ED. 37.4% of participants visited the ED during the containment phase, 40.4% during the transition phase, and 22.2% in the mitigation phase. Almost half of ED attendees in the containment phase were not expecting any diagnostic test (50.7%), while half (49.0%) in the transition phase expected a COVID-19 test during their ED visit.

Of the enabling factors for which participants visited the ED for URTI, 28.6% indicated that their employer would cover their bill, 44.1% indicated that they were adhering to their employer's health policy, 23.2% were referred by a healthcare provider, and 68.4% cited convenience. Significantly lower proportions of participants not expecting any diagnostic tests in the ED visited the ED in accordance with their employer's health policy (16.4%) or had their hospital bill covered by their employer (16.4%) (*p* < 0.001 for both), while a significantly higher proportion of those expecting non-COVID-19-specific diagnostic tests (39.2%) were referred to the ED by other healthcare providers (*p* < 0.001).

#### Individual needs

Of participants visiting the ED to fulfill individual needs, 39.1% cited having persistent symptoms or illness, 42.4% thought that their illness was severe, and 30.8% were worried about contracting COVID-19. Most participants (90.8%) were satisfied with their ED visit. A significantly higher proportion (39.5%) of participants worried about contracting COVID-19 were expecting both COVID-19-specific and non-COVID-specific diagnostic tests (*p* < 0.001), while a significantly lower proportion (32.4%) of participants who perceived their illness as severe expected only a COVID-19 diagnostic test.

### Determinants of expecting a diagnostic test in the ED during the COVID-19 pandemic

#### Expect only a COVID-19-specific test

##### Predisposing factors

Participants aged 21–40 were almost three times (adjusted odds ratio (aOR): 2.98, 95% confidence interval (CI) [1.04–8.55]) as likely as those aged above 60 to expect a COVID-19-specific diagnostic test during their ED visit (Table 3). Those without a prior clinical consultation for the same illness were also twice (2.10 [1.13–3.89]) as likely to expect a COVID-19-specific diagnostic test during their visit.

**Table 3:** Multinomial logistic regression of expectation for ED diagnostic services.

**Andersen's category**	**Model variables**	**Expects a COVID-19-specific test**	**Expects a non-COVID-19-specific test**	**Expects a COVID-19-specific + non-COVID-19-specific tests**	**VIF**
	**(Base: Not expecting any tests)**	**Adjusted OR (95% CI)**	**Adjusted OR (95% CI)**	**Adjusted OR (95% CI)**	
	**Age**				
Predisposing	Aged above 60	Ref	Ref	Ref	
	Aged 41–60	2.35 (0.73, 7.60)	0.92 (0.28, 2.99)	4.49 (1.43, 14.13)^*^	3.66
	Aged 21–40	2.98 (1.04, 8.55)^*^	0.89 (0.32, 2.49)	3.61 (1.26, 10.38)^*^	3.50
	**Prior (non-ED) healthcare consult for URTI**				
Predisposing	No prior consult	2.10 (1.13, 3.89)^*^	0.89 (0.44, 1.79)	1.12 (0.62, 2.03)	1.20
	**Employment**				
Enabling	Employed	Ref	Ref	Ref	
	Not employed	2.38 (0.79, 7.20)	3.83 (1.26, 11.66)^*^	1.74 (0.60, 5.08)	3.21
	Full-time national servicemen	1.99 (0.76, 5.25)	0.33 (0.06, 1.81)	0.85 (0.31, 2.37)	3.40
	**Payment method**				
Enabling	Out-of-pocket	Ref	Ref	Ref	
	Employee benefits	1.80 (0.92, 3.52)	1.06 (0.48, 2.36)	1.46 (0.76, 2.81)	1.60
	Government/private insurance	0.85 (0.31, 2.31)	0.84 (0.29, 2.44)	1.18 (0.47, 2.95)	1.20
	Social subsidies	0.36 (0.04, 3.20)	0.24 (0.02, 3.11)	0.64 (0.09, 4.47)	1.12
	**Employers' policy**				
Enabling	Adheres to employers' policy	3.70 (1.79, 7.67)^**^	1.95 (0.80, 4.79)	2.94 (1.41, 6.14)^*^	1.12
	**Perception of illness severity**				
Individual need	Thinks that illness is severe	0.40 (0.22, 0.72)^*^	1.36 (0.68, 2.70)	0.83 (0.47, 1.47)	1.13
	**Concerns about contracting COVID-19**				
Individual need	Worried about contracting COVID-19	2.29 (1.11, 4.69)^*^	0.51 (0.19, 1.36)	2.95 (1.45, 5.99)^*^	1.32
	**COVID-19 phases**				
	Containment	Ref	Ref	Ref	
	Transition	2.29 (1.15, 4.56)^*^	1.37 (0.60, 3.16)	2.03 (1.02, 4.06)^*^	1.34
	Mitigation	1.04 (0.51, 2.11)	0.96 (0.42, 2.18)	2.02 (1.03, 3.97)^*^	1.30

^*^p < 0.05, ^**^p < 0.001.

VIF, Variance inflation factor.

##### Enabling factor

Participants adhering to their employer's health policy to visit the ED for suspected COVID-19 were 3.7 times (3.70 [1.79–7.67]) as likely to expect only a COVID-19-specific diagnostic test.

##### Individual needs

Participants who thought their illness was not severe were 2.5 times (2.50 [1.39–4.55]) as likely as those who perceived their illness to be severe to expect a COVID-19-specific diagnostic test. Those worried about contracting COVID-19 were also 2.3 times (2.29 [1.11–4.69]) as likely to expect a COVID-19-specific diagnostic test.

##### COVID-19 phases

Compared with the containment phase, participants in the transition phase were 2.3 times (2.29 [1.15–4.56]) as likely to expect only a COVID-19-specific diagnostic test.

#### Expect a non-COVID-19-specific test

##### Enabling factor

Non-employed participants were 3.8 times (3.83 [1.26–11.66]) as likely as employed participants to expect non-COVID-19-specific diagnostic tests. All other factors were not statistically significant.

#### Expect both COVID-19-specific and non-COVID-19-specific tests

##### Predisposing factor

Participants aged 21–40 and 41–60 were 3.6 (3.61 [1.26–10.38]) and 4.5 times (4.49 [1.43–14.13]) as likely as adults above 60 years old to expect both COVID-19-specific and non-COVID-19-specific diagnostic tests during their ED visit (Table 3).

##### Enabling factor

Participants adhering to their employer's health policy to visit the ED for suspected COVID-19 were almost three times (2.94 [1.41–6.14]) as likely to expect both COVID-19-specific and non-COVID-19-specific diagnostic tests.

##### Individual needs

Participants were almost three times (2.95 [1.45– 5.99]) as likely to expect both COVID-19-specific and non-COVID-19-specific diagnostic tests if they were worried about contracting COVID-19.

##### COVID-19 phases

Compared with the containment phase, participants in the transition (2.03 [1.02–4.06]) and mitigation (2.02 [1.03–3.97]) phases were twice as likely to expect both COVID-19-specific and non-COVID-19 specific diagnostic tests during their ED visit.

### Reasons for patients with URTI to visit the ED

**Figure 1:**
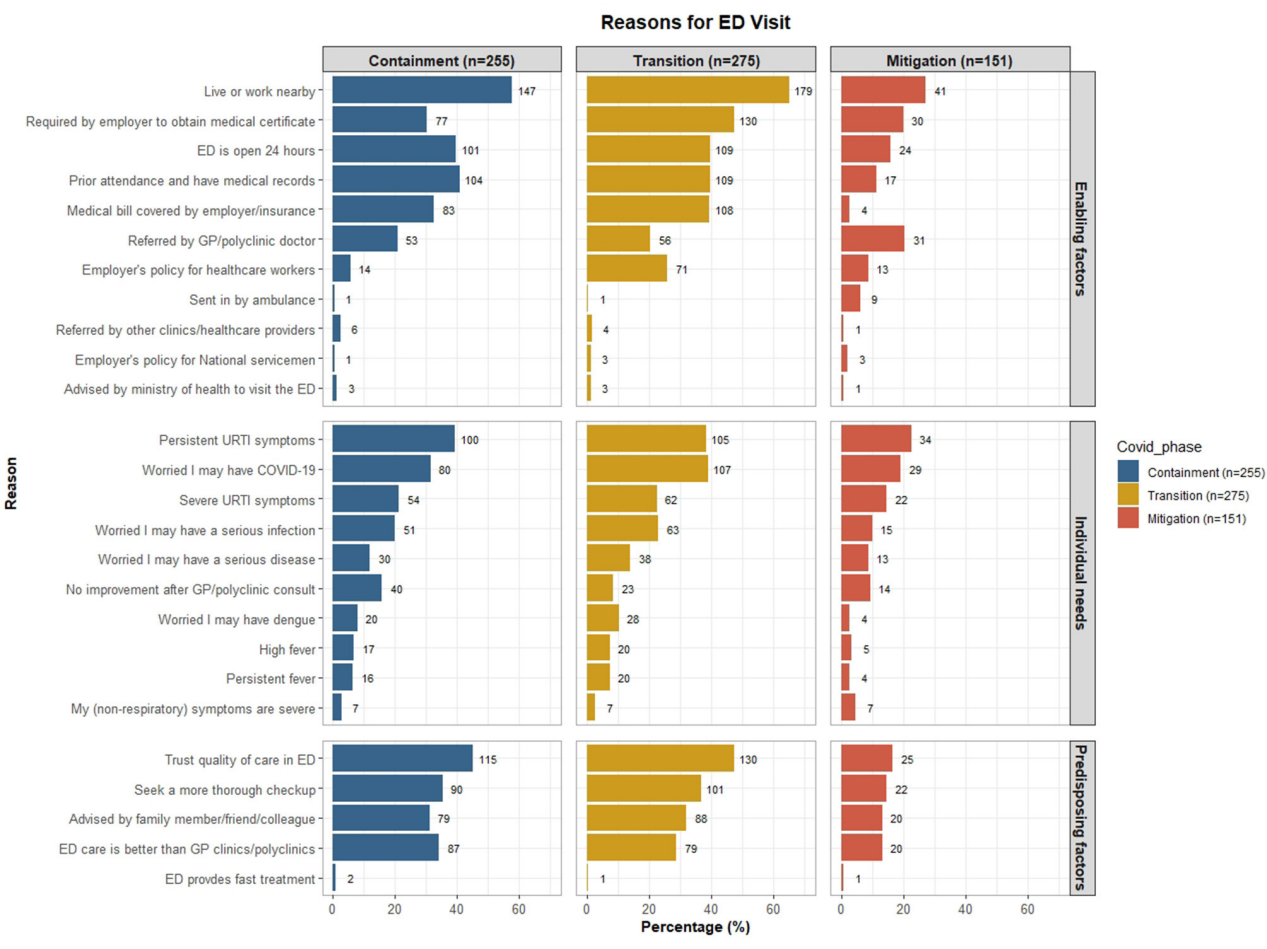
Reasons for visiting the ED during different phases of the COVID-19 pandemic, grouped by the three determinant components of Andersen's healthcare utilization model. Participants can list more than one reason.

Figure 1 shows the reasons for visiting the ED during different phases of the COVID-19 pandemic. The reasons are grouped by the three determinant components of Andersen's healthcare utilization model. A larger proportion of participants reported more reasons during the containment and transition phase of COVID-19 compared with the mitigation phase.

The top enabling factor for visiting the ED for URTI throughout all phases of the COVID-19 pandemic is the convenience of living or working near the ED. More than half of the participants cited this reason during the containment (57.6%) and transition (65.1%) phases, while 27.1% cited this reason during the mitigation phase. Similarly, more participants cited a “requirement for obtaining a medical certificate” and healthcare financing (i.e., bill covered by employer or insurance) during the containment and transition phase compared with the mitigation phase. The proportion of participants citing “bill covered by their employer or insurance” was notably lower during the mitigation phase (2.6%) compared with the other phases (~30– 40%). A fifth of participants cited that they were “referred to the ED by a primary care doctor” consistently over the three COVID-19 phases, while a quarter of them (25.8%) cited “employer's policy for healthcare workers to visit the ED for URTI symptoms” during the transition phase.

The perceived need to visit the ED did not vary too much across the COVID-19 phases, except that the worry of contracting COVID-19 was more pronounced during the transition phase. Reasons such as having “persistent URTI symptoms” and “worried about contracting COVID-19” were the top two perceived needs for visiting the ED.

The top predisposing factor for visiting the ED during all phases of the COVID-19 pandemic is the trust in the quality of care the ED provides. This reason was pronounced during the containment and transition phase compared with the mitigation phase (>40% vs. 16.5%). Other notable reasons include a desire to “seek a more through check-up”, “advised by friends/ family/ colleagues” to visit the ED and thinks that “ED care is better than primary care”.

## Discussion

Our study has provided invaluable insights into patients' changing expectations for diagnostic tests during ED visits for uncomplicated URTI during the COVID-19 pandemic. Applying Andersen's healthcare utilization model enabled us to ascertain factors influencing patient expectations for ED visits, which would guide future pandemic preparedness planning in preventing the overwhelming use of ED resources. We observed that being younger and not having a prior medical consultation for URTI in other healthcare institutions were significant predisposing factors influencing expectations for a COVID-19-specific diagnostic test. Enabling factors for expecting a COVID-19-specific diagnostic test include adherence to employment policy and the COVID-19 pandemic phase, while individual needs include non-perception of illness severity and worry about contracting COVID-19.

Adults aged 60 years and below were more likely than their older counterparts to expect a COVID-19-specific test or a combination of COVID-19-specific and non-COVID-19-specific diagnostic tests during their ED visit for uncomplicated URTI. There is growing evidence of young adults having disproportionately higher non-urgent ED visits (3). Studies in other countries have found that convenience, concern about the seriousness of symptoms and desire for reassurance, influence from friends and family, and negative perceptions about alternatives, such as primary care providers, are reasons young adults seek non-urgent ED care (3). We found similar reasons, such as the desire to seek a more thorough check-up, being advised by friends, family, or colleagues to visit the ED, and thinking that ED care is better than primary care. We also found that the top predisposing reason for visiting the ED during the COVID-19 pandemic was trust in the quality of ED care. With the ED equipped with services that many primary care facilities lack, such as X-ray and blood test services (24), patients can receive a more thorough examination by visiting the ED. As such, patients may deem ED services superior to primary care facilities (25), which likely contributed to the surge of non-urgent ED visits for URTI during the containment and transition phases of the COVID-19 pandemic in Singapore.

We found that non-employed participants were 3.8 times more likely to expect non-COVID-19-specific diagnostic tests during their ED visit. These non-employed participants were likely retired older adults presenting to the ED with non-specific symptoms that are hard to differentiate from acute symptoms (26). Some patients may prefer specialist care and easy access to radiologic and laboratory diagnostic tests in a one-stop center such as an ED (27). Furthermore, patients may also perceive the all-inclusive (medical consultation and diagnostic tests) fixed ED cost to be lower than the combined charges of consultation fees and individual diagnostic tests in primary care clinics.

The employer's health policy was a significant enabling factor for patients expecting only COVID-19-specific tests and both COVID-19-specific and non-COVID-19-specific diagnostic tests at the ED. This finding was unsurprising as newly devised URTI protocols for healthcare workers encouraged them to seek care at their respective institutions if they experienced URTI symptoms, for which employee benefits covered the costs. Hence, the convenience of accessing ED services and reduced medical fees in the form of employee benefits, insurance, or subsidies made visiting the ED a natural choice for many healthcare workers (3, 25).

Expectations for ED diagnostic tests were dynamic across the COVID-19 pandemic phases. Twice as many participants expected either only a COVID-19-specific diagnostic test or both COVID-19-specific and non-COVID-19-specific tests during the transition phase compared with the containment phase. However, the expectation for both COVID-19-specific and non-COVID-19-specific diagnostic tests remained twice as high during the mitigation phase as the containment phase. Notably, the expectation for only a COVID-19-specific diagnostic test was similar between the mitigation and containment phases. The change in expectations regarding COVID-19-specific tests in the mitigation phase is likely due to the availability of COVID-19 self-test kits in the community, which became more readily available with the nationwide distribution of the kits to every household by the Singaporean government.

As anticipated, patients worried about contracting COVID-19 were more likely to expect a COVID-19-specific diagnostic test. However, those who thought that their illness was severe were less likely to expect any diagnostic test. This observation could be attributed to the wide range of patient expectations when seeking emergency care. Patients may have greater expectations for the care and medical services of the ED visit beyond mere diagnostics services (28, 29). As evident from our findings, patients have many reasons for visiting the ED, such as having persistent URTI symptoms and worries about a severe infection. These individual needs exist across the COVID-19 pandemic phases and may not be related to the pandemic. Therefore, unnecessary ED visits may persist even after the COVID-19 pandemic, and broader considerations are required to design interventions for mitigating inappropriate ED use.

The strengths of our study include measuring the changes in the population's healthcare-seeking behavior over the various phases of the pandemic and surveying actual URTI patients in the ED to obtain real-world responses. To the best of our knowledge, this is the first study analyzing the reasons behind ED visits in patients with uncomplicated URTI during the pandemic, and the findings would be useful for addressing gaps in public health communications during the pandemic. However, our study is limited by not assessing the arrival time of patients attending the ED. Patients visiting the ED after office hours may have different reasons for making non-urgent ED visits and hence have differing expectations in the ED. There may also be recall bias as this was a self-reported cross-sectional study, although the bias was likely to be minimal as all participants completed the survey at the ED visit. We could not comprehensively assess the motivations for which participants attended the ED, which could shed light on other changing needs across the pandemic phases. Future studies could take a mixed-methods approach to enhance the interpretation of our findings.

Despite the milder disease presentation of COVID-19 in a highly vaccinated population and the government's encouragement of people with mild COVID-19 symptoms to visit primary care clinics, individuals with URTI symptoms continue to visit the ED with the expectation of receiving a COVID-19 diagnostic test during the mitigation phase. These patients could be confused about the dynamic national COVID-19 policies communicated to them over the course of the evolving pandemic (30). Although the government's communications on COVID-19 measures were frequent and transparent, their approach toward managing the pandemic was reactive rather than proactive (31). The constantly changing rules on safe management measures inadvertently caused confusion and significant anxiety among the public (30). Therefore, besides redirecting patients to primary care facilities such as public health preparedness clinics, strengthening the healthcare system's adaptability to changes and improving public communications are essential measures to enhance national preparedness for the next pandemic. For instance, tailoring public communication strategies to the different pandemic phases, such as encouraging young people to seek care at primary care clinics during the transition phase of the pandemic, might help to divert resources to those who need the service more.

## Conclusion

In conclusion, patients' expectations for diagnostic tests during ED visits for uncomplicated URTI were dynamic across the COVID-19 pandemic phases. With the widespread availability of self-test kits during the mitigation phase, expectations for diagnostic tests shifted to having both COVID-19-specific and non-COVID-19-specific tests. Expectations were also higher among younger individuals and those worried about contracting COVID-19 during the pandemic. Enabling factors such as convenience, the requirement to obtain a medical certificate, and adherence to employers' health policy for URTI were pronounced during the transition phase. Other factors, including the public's perception of better care for URTI at EDs than at primary care clinics, were beyond the pandemic and should be addressed post-pandemic. Future work is required to enhance public communications on the availability of diagnostic services in primary care and public education on self-management of emerging infectious diseases such as COVID-19.

## Data availability statement

The datasets presented in this article are not readily available because the data contains sensitive information. Requests to access the datasets should be directed to the corresponding author.

## Ethics statement

The studies involving humans were approved by National Healthcare Group Domain Specific Review Board in Singapore. The studies were conducted in accordance with the local legislation and institutional requirements. The participants provided their written informed consent to participate in this study.

## Author contributions

ZH contributed to conceptualization, methodology, formal analysis, data curation, writing—original draft, project administration, and supervision. KN contributed to data collection, writing—original draft, and writing—review and editing. WK, HL, YW, and HT contributed to project administration and writing—review and editing. ES, JO, and LP contributed to writing—review and editing. AC contributed to conceptualization, methodology, supervision, funding acquisition, and writing—review and editing. All authors contributed to the article and approved the submitted version.
